# Multicenter evaluation of a digital screening tool for early identification of temporomandibular disorder risk in Colombian and Mexican adults

**DOI:** 10.3389/fmed.2026.1794091

**Published:** 2026-04-10

**Authors:** Irene Aurora Espinosa De Santillana, Olga Patricia López-Soto, Juan Alberto Aristizábal-Hoyos, Héctor Fuentes-Barria, Raúl Aguilera-Eguía, Andrea Quintero-Sánchez, Valentina Estrada-Ramírez, Mónica Johana Peña-Arisitizábal, Ángel Roco-Videla

**Affiliations:** 1 Departamento de Cirugía, Facultad de Estomatología, Benemérita Universidad Autónoma de Puebla, Puebla, Mexico; 2 Departamento de Salud Oral, Facultad de Salud, Universidad Autónoma de Manizales, Manizales, Colombia; 3 Centro de Investigación en Medicina de Altura (CEIMA), Universidad Arturo Prat, Iquique, Chile; 4 Departamento de Salud Pública, Facultad de Medicina, Universidad Católica de la Santísima Concepción, Concepción, Chile; 5 Residentes Posgrado de Rehabilitación Oral, Facultad de Salud, Universidad Autónoma de Manizales, Manizales, Colombia; 6 Dirección de Desarrollo y Postgrado, Universidad Autónoma de Chile, Santiago, Chile

**Keywords:** bruxism, dentristry, mobile applications, risk factors, temporomandibular joint disorders

## Abstract

**Objective:**

To evaluate the performance, applicability, and real-world utility of a validated digital screening tool for early identification of temporomandibular disorder (TMD) risk in Colombian and Mexican adults.

**Methods:**

A cross-sectional, multicenter study included 429 adults (299 Mexico, 130 Colombia) who completed a standardized digital application assessing joint pain, bruxism, mandibular locking, joint sounds, parafunctional habits, trauma history, and emotional factors. TMD risk was classified using a composite digital score into low, moderate, or high categories, emphasizing that the tool measures participants’ risk classification and internal construct consistency rather than population prevalence or causal risk factors. Descriptive statistics (mean ± SD, frequencies %) and effect sizes (Cohen’s d, Cramér’s V) were calculated. Logistic regression identified independent predictors of high TMD risk.

**Results:**

Mean age was 29.9 ± 13.3 years in Mexico and 28.8 ± 10.8 in Colombia (*p* = 0.35, *d* = 0.09). Diurnal bruxism (OR = 12.43), joint pain (OR = 7.73), mandibular locking (OR = 4.98), other parafunctional habits (OR = 4.61), nocturnal bruxism (OR = 3.34), fatigue during mastication (OR = 2.84), history of trauma (OR = 3.40), and female sex (OR = 3.42) were independent predictors of high TMD risk, demonstrating the tool’s capability to identify key risk patterns consistently across countries.

**Conclusion:**

The digital screening tool effectively stratified TMD risk in a multicenter, real-world setting, highlighting its potential for early identification, population-based screening, and clinical implementation in diverse Latin American populations. Indicators classified as high-risk were observed more frequently among women and younger adults.

## 1 Introduction

Temporomandibular disorders (TMD) represent a heterogeneous and complex group of musculoskeletal pain syndromes affecting the temporomandibular joint (TMJ) and associated musculature, constituting a growing public health concern worldwide (1, 2). Their most frequent clinical manifestations include pain in the TMJ region and/or masticatory muscles, joint sounds, and limited mandibular movements, often extending to headaches, otalgia, and cervical pain. In more severe cases, TMD significantly and persistently impacts health-related quality of life, driving the need for specialized healthcare attention (3, 4).

Despite their high prevalence and morbidity burden, TMD diagnosis remains a considerable clinical challenge. Its multifactorial nature requires comprehensive assessment by orofacial pain and dental specialists, limiting timely identification and management in many healthcare settings (5, 6). This challenge is further compounded by the global shortage of specialists; however, according to current DC/TMD standards, accurate diagnosis relies primarily on clinical examination and patient history, with imaging reserved for selected cases (6, 7). Moreover, standardized diagnostic systems, such as the DC/TMD, although robust and validated, may be complex, time-consuming, and impractical for large-scale application outside specialized clinical environments (8).

Recent evidence indicates that risk of TMD, as measured by population-based screening tools, affects approximately 29%–34% of adults, with particularly high figures in populations exposed to elevated psychosocial stress and parafunctional habits (1, 2). Population-based studies and global analyses suggest that TMD risk is prevalent among young and economically active populations, with projections indicating a potential increase up to 44% by 2050 (9).

Global analyses suggest that TMD risk is prevalent among young and economically active populations (1, 2), with individual studies in Asian populations, such as Chinese university students, reporting rates near 31% (9), whereas in Latin America, estimates are around 35% (10). Importantly, more severe forms of TMD are associated with substantial deterioration of oral health-related quality of life, emphasizing the need for strategies that enable early identification and prioritization of high-risk individuals (11).

This high epidemiological burden, combined with marked heterogeneity in clinical presentation and disease severity, is largely explained by the multifactorial etiology of TMD, which includes oral parafunctional habits (such as bruxism, unilateral chewing, and tooth clenching), psychological factors (stress, anxiety, and depression), occlusal alterations, and a close association with primary headaches, including migraine and tension-type headache (1, 2). Such complexity challenges traditional case-finding approaches and underscores the need for accessible, standardized, and easily deployable screening tools capable of stratifying TMD risk rather than providing a clinical diagnosis.

In recent years, digital health solutions, including mobile applications, have emerged as a promising alternative to support population-based screening and initial risk assessment for complex conditions. These tools can expand access to care, optimize healthcare resource allocation, and reduce costs associated with delayed diagnosis or specialist referral (12, 13). Despite the proliferation of digital screening tools, there remains limited multicenter, real-world evidence evaluating their performance, applicability, and capacity to stratify TMD risk across diverse sociocultural contexts, particularly in Latin American populations.

Therefore, the objective of the present study was not only to assess the distribution of TDM risk using a previously validated digital application and provide multicenter evidence on its real-world performance as an early risk stratification tool, focusing on classification of participants’ risk rather than estimation of prevalence in adult populations from Colombia and Mexico.

## 2 Materials and methods

### 2.1 Design

This was an observational, cross-sectional, multicenter international study conducted in Colombia and Mexico, corresponding to the second phase of a research project aimed at validating and implementing a digital TMD risk screening tool. The protocol was initially approved by the Ethics Committee of the Autonomous University of Puebla (Protocol Code: 2024-2108-121). All procedures adhered to the principles of the Declaration of Helsinki and were reported in accordance with the STROBE (Strengthening the Reporting of Observational Studies in Epidemiology) guidelines to ensure methodological rigor and transparency in observational research (14, 15). Although recruitment was conducted via a mobile application and not through physical clinical centers, the study provides multicenter evidence by including participants from multiple geographic regions.

### 2.2 Context

Early identification of temporomandibular disorders in non-specialized settings is limited due to the complexity of clinical diagnosis and challenges in applying standardized diagnostic systems in population-based, multicenter studies. This restricts accurate estimation of TMD risk distribution outside specialized clinical environments. The study is intended as a screening assessment rather than a diagnostic investigation, emphasizing classification of TMD risk rather than confirmed prevalence.

In this context, the use of a mobile digital application to predict TMD risk, developed by the Autonomous University of Puebla^1^, represents an accessible, standardized, and scalable alternative. Prior validation demonstrated a sensitivity of 92.3% and specificity of 60%, which indicates a risk of false positives. This limitation is acknowledged and discussed in the limitations section. Positive predictive value of 85.7%, and moderate agreement with DC/TMD criteria (kappa = 0.56) (16). This study provides external, real-world evidence by applying this validated tool in a multicenter setting, supporting its potential use beyond the original validation environment.

### 2.3 Participants

The sample consisted of 429 participants (130 from Colombia and 299 from Mexico), selected via non-probabilistic convenience sampling, who voluntarily agreed to participate. Inclusion criteria were: age ≥ 18 years; residence in Colombia or Mexico; basic familiarity with a smartphone running Android or iOS; and informed consent. Exclusion criteria included physical, sensory, or technological limitations preventing proper use of the mobile device, as well as neurological or cognitive disorders that could impair comprehension or accurate responses to the instrument.

Convenience sampling was deemed appropriate for exploratory, implementation-oriented assessment of the tool’s real-world applicability; however, this introduces potential selection bias and limits generalizability. These limitations are acknowledged and discussed.

### 2.4 Clinical data collection

Clinical data were collected using a standardized, secure, and accessible digital platform. After recruitment, participants downloaded the mobile application on their personal devices through a secure link provided via institutional email or official social media channels. The application guided each user through registration, digital informed consent, and completion of the structured questionnaire in a stepwise and user-friendly manner. Procedures for data quality control were applied, including real-time validation checks and automated alerts for missing or inconsistent responses, to minimize information bias.

### 2.5 TMD risk assessment

Temporomandibular disorder risk was estimated using a 10-item dichotomous questionnaire evaluating key clinical indicators, including mandibular or facial pain, joint sounds, functional limitation, bruxism, improper tooth use, history of mandibular trauma, and psycho-emotional factors. Responses were recorded directly by participants and transmitted automatically to a central database in real time, ensuring consistency and data security. Each item was weighted according to clinical relevance, allowing classification of TMD risk into low, moderate, or high levels (16).

Importantly, the resulting TMD risk score reflects a composite screening construct rather than a definitive clinical diagnosis and is intended solely for early risk stratification. It is explicitly acknowledged that symptoms such as bruxism, joint clicking, or jaw locking require clinical confirmation, and self-assessment may introduce information bias.

### 2.6 Sociodemographic and clinical variables

Demographic data included age (years), biological sex (male/female), and country of residence (17). Clinical variables encompassed TMD-associated indicators such as mandibular/facial pain, fatigue during mastication, mandibular locking, joint sounds, daytime and nighttime bruxism, morning stiffness, improper tooth use, trauma history, and psychosocial factors. Emotional factors were assessed using a simplified questionnaire capturing negative emotional states in the previous month, following the general recommendations of DC/TMD, but not as a full-scale psychosocial assessment (18, 19).

This contextualizes the robustness of the observed estimates and clarifies that these measures do not replace an *a priori* sample size calculation.

### 2.7 Sample size and statistical power

Sample selection was performed via non-probabilistic convenience sampling. Although a *post hoc* statistical power calculation was conducted using G*Power 3.1.9.7 (20), it does not replace an *a priori* sample size calculation. A one-sample binomial test was applied to evaluate the observed proportion of participants classified as high-risk (27%) relative to a reference value from a previous study (16%) in Peruvian adults aged 18–62 years, used here only as a comparison benchmark rather than an estimate of population prevalence (21). Using an effect size of 0.28, a significance level of α = 0.05, and a sample size of 429 participants, the *post hoc* power was approximately 99%, exceeding the conventional threshold of 80% necessary for detecting differences in high-risk classification (20).

### 2.8 Bias

Several potential sources of bias inherent to the study design were considered. First, selection bias may be present due to non-probabilistic convenience sampling, smartphone requirements, and voluntary participation, which could limit representativeness and generalizability and potentially exclude individuals with limited digital literacy or lower socioeconomic status (22). Geographic restriction of the sample may further influence external validity, as participants were recruited from specific regions within Mexico and Colombia (23). Future implementations may mitigate this bias by offering alternative access methods for participants without smartphones.

Second, although key variables were assessed using standardized and validated instruments, small sources of information bias may persist due to individual interpretation, recall bias, or cultural differences between study regions (24). Self-reported data, including bruxism, joint clicking, or jaw locking, introduce a potential for misclassification; this limitation is explicitly acknowledged. Data collection via a digital platform minimizes interviewer bias and ensures consistency. Finally, the cross-sectional design limits causal inference, and potential confounders were addressed as much as possible through validated measures and standardized procedures (25). All methodological limitations, including the specificity of the screening tool, *post hoc* power analysis, absence of clinical verification, and multicenter designation based on digital recruitment, are now explicitly discussed to provide transparency and contextualize interpretation of the results.

### 2.9 Statistical analysis

Data were analyzed using SPSS version 27 (IBM Corp., Armonk, NY, USA). Continuous variables were summarized as mean ± standard deviation (SD) with 95% confidence intervals (CI), while categorical variables were presented as frequencies and percentages. Normality of continuous variables was assessed using the kolmogorov smirnov test.

For comparisons between two independent groups (men vs. women), Student’s *t*-tests were applied only to the aged for normally distributed variables with homogeneity of variances (evaluated by Levene’s test) (26, 27). Categorical variables were compared using Pearson’s chi-square tests (28). Appropriate effect sizes were reported for all inferential analyses: Cohen’s d for *t*-tests, r for Mann–Whitney U tests, and Cramér’s V for chi-square tests (29, 30). Effect sizes were interpreted with thresholds appropriate for dental research (r: 0.20 small, 0.40 medium, 0.70 large; d/Hedges g: 0.10 small, 0.40 medium, 0.90 large), in accordance with contemporary methodological literature, rather than using classical Cohen thresholds (31).

To explore factors associated with high TMD risk, logistic regression analyses were performed multivariable model was adjusted for sex, age, and country and included joint pain, fatigue on mastication, mandibular locking, joint sounds, nocturnal bruxism, diurnal bruxism, morning fatigue, other parafunctional habits, emotional factors, and history of trauma. Results were expressed as adjusted odds ratios (OR) with 95% confidence intervals (95% CI).

Multicollinearity among independent variables was assessed prior to model fitting. Model calibration was evaluated using the Hosmer–Lemeshow goodness-of-fit test. Statistical significance was set at a two-tailed α level of 0.05.

## 3 Results

### 3.1 Sociodemographic characteristics

The study population consisted of 429 participants, including 299 from Mexico and 130 from Colombia. The mean age was 29.91 ± 13.27 years in Mexico and 28.76 ± 10.81 years in Colombia, with no statistically significant difference between the two countries (*p* = 0.35; Cohen’s *d* = 0.09). Similarly, no significant age differences were observed between men (28.66 ± 12.17 years) and women (30.04 ± 12.78 years; *p* = 0.28; Cohen’s *d* = 0.11). When stratified by WHO age groups (18–44, 45–59, ≥60 years), the majority of participants in both countries and both sexes were in the 18–44 years category, and no statistically significant differences were found in the distribution of age groups between countries (*p* = 0.16; effect size = 0.09) or between sexes (*p* = 0.41; effect size = 0.07). These findings indicate a high degree of homogeneity and comparability across countries and sexes (Table 1).

**TABLE 1:** Sociodemographic characteristics of the study population (*n* = 429), stratified by country and sex.

Variable		Mexico (*n* = 299)	Colombia (*n* = 130)	*P*-value	Effect size	Male (*n* = 148)	Female (*n* = 281)	*P*-value	Effect size
Age (years)		29.91 ± 13.27	28.76 ± 10.81	0.35	0.09	28.66 ± 12.17	30.04 ± 12.78	0.28	0.11
Age group (WHO)	18–44	243 (81.27)	115 (88.46)	0.16	0.09	128 (86.49)	230 (81.85)	0.41	0.07
	45–59	41 (13.71)	12 (9.23)			14 (9.46)	39 (13.88)		
	≥60	15 (5.01)	3 (2.31)			6 (4.05)	12 (4.27)		

### 3.2 Clinical characteristics

Regarding clinical indicators and risk factors, mandibular locking was significantly more prevalent in Mexico than in Colombia (21.7% vs. 8.5%; *p* = 0.001; *V* = 0.16), and other parafunctional habits were also higher in Mexico (47.2% vs. 35.4%; *p* = 0.02; *V* = 0.11). No significant differences were observed between countries for joint pain, joint sounds, diurnal bruxism, nocturnal bruxism, morning fatigue, emotional factors, or history of trauma.

When stratified by sex, women reported higher proportions of several clinical indicators: joint pain (44.8% vs. 31.8%; *p* = 0.006; *V* = 0.13), fatigue during mastication (52.7% vs. 39.2%; *p* = 0.007; *V* = 0.13), nocturnal bruxism (54.5% vs. 41.9%; *p* = 0.01; *V* = 0.12), morning fatigue (46.6% vs. 22.3%; *p* = 0.001; *V* = 0.24), history of trauma (12.5% vs. 37.8%; *p* = 0.001; *V* = 0.30), and emotional factors (67.3% vs. 56.8%; *p* = 0.03; *V* = 0.10). No significant sex differences were observed for mandibular locking, joint sounds, or diurnal bruxism (V ≤ 0.03), highlighting patterns within the study cohort rather than implying population-level differences.

The combined TMD risk classification showed similar proportions of high-risk participants between Mexico and Colombia (32.8% vs. 29.2%; *p* = 0.50; *V* = 0.09), whereas women had a higher proportion of high-risk classification compared to men (37.7% vs. 20.3%; *p* = 0.001; *V* = 0.18). These results indicate that the digital tool consistently identifies expected TMD risk patterns across sexes and geographic contexts in this multicenter sample (Table 2).

**TABLE 2:** Clinical characteristics of the study population (*n* = 429), stratified by country and sex.

Variable		Mexico (*n* = 299)	Colombia (*n* = 130)	*P*-value	Effect size	Male (*n* = 148)	Female (*n* = 281)	*P*-value	Effect size
Joint pain	Yes	125 (41.81)	48 (36.92)	0.34	0.05	47 (31.76)	126 (44.84)	0.006	0.13
	No	174 (58.19)	82 (63.08)			101 (68.24)	155 (55.16)		
Fatigue on mastication	Yes	137 (45.82)	69 (53.08)	0.17	0.07	58 (39.19)	148 (52.67)	0.007	0.13
	No	162 (54.18)	61 (46.92)			90 (60.81)	133 (47.33)		
Mandibular locking	Yes	65 (21.74)	11 (8.46)	0.001	0.16	26 (17.57)	50 (17.79)	0.95	0.003
	No	234 (78.26)	119 (91.54)			122 (82.43)	231 (82.21)		
Joint sounds	Yes	150 (50.17)	65 (50)	0.97	0.002	71 (47.97)	144 (51.25)	0.52	0.03
	No	149 (49.83)	65 (50)			77 (52.03)	137 (48.75)		
Nocturnal bruxism	Yes	157 (52.51)	58 (44.62)	0.13	0.07	62 (41.89)	153 (54.45)	0.01	0.12
	No	142 (47.49)	72 (55.38)			86 (58.11)	128 (45.55)		
Morning fatigue	Yes	120 (40.13)	44 (33.85)	0.22	0.06	33 (22.30)	131 (46.62)	0.001	0.24
	No	179 (59.87)	86 (66.15)			115 (77.70)	150 (53.38)		
Diurnal bruxism	Yes	120 (40.13)	54 (41.54)	0.79	0.01	57 (38.51)	117 (41.64)	0.53	0.03
	No	179 (59.87)	76 (58.46)			91 (61.49)	164 (58.36)		
Other parafunctional habits	Yes	141 (47.16)	46 (35.38)	0.02	0.11	61 (41.22)	126 (44.84)	0.47	0.04
	No	158 (52.84)	84 (64.62)			87 (58.78)	155 (55.16)		
History of trauma	Yes	67 (22.41)	24 (18.46)	0.36	0.04	56 (37.84)	35 (12.46)	0.001	0.30
	No	232 (77.59)	106 (81.54)			92 (62.16)	246 (87.54)		
Emotional factors	Yes	197 (65.88)	76 (58.46)	0.14	0.07	84 (56.76)	189 (67.26)	0.03	0.10
	No	102 (34.12)	54 (41.54)			64 (43.24)	92 (32.74)		
TMD risk (combined)	High	98 (32.78)	38 (29.23)	0.50	0.09	30 (20.27)	106 (37.72)	0.001	0.18
	Moderate	122 (40.80)	61 (46.92)			71 (47.97)	112 (39.86)		
	Low	79 (26.42)	31 (23.85)			47 (31.76)	63 (22.42)		

### 3.3 Multivariable logistic regression analysis

Multivariable logistic regression analysis was performed to evaluate the internal construct validity and consistency of the digital screening tool, rather than to identify causal or etiological risk factors. Several clinical indicators were associated with high-risk classification: diurnal bruxism showed the strongest association (adjusted OR = 12.43; 95% CI: 5.97–25.89; *p* < 0.001), followed by joint pain (OR = 7.73; 95% CI: 3.76–15.92; *p* < 0.001), mandibular locking (OR = 4.98; 95% CI: 1.99–12.41; *p* = 0.001), other parafunctional habits (OR = 4.61; 95% CI: 2.26–9.43; *p* < 0.001), nocturnal bruxism (OR = 3.34; 95% CI: 1.59–7.03; *p* = 0.002), fatigue during mastication (OR = 2.84; 95% CI: 1.46–5.51; *p* = 0.002), and history of trauma (OR = 3.40; 95% CI: 1.40–8.30; *p* = 0.007). Female sex was also associated with high-risk classification (OR = 3.42; 95% CI: 1.47–7.99; *p* = 0.004). Age, country of residence, morning fatigue, and emotional factors were not independently associated after adjustment (*p* > 0.05). These associations should not be interpreted as causal relationships.

As the outcome variable was derived from a composite screening score, these associations should be interpreted as evidence of internal construct validity and consistency of the digital TMD risk tool rather than as causal relationships. The magnitude of the observed odds ratios reflects the relevance of each clinical indicator within the risk score, supporting the tool’s capacity to accurately stratify participants according to TMD risk in a real-world, multicenter context (Table 3).

**TABLE 3:** Multivariable logistic regression analysis for TMD risk high.

Variable	B	SE	Wald	Adjusted OR	95% CI	*P*-value
Sex	1.23	0.43	8.09	3.42	1.47–7.99	0.004
Country	0.49	0.37	1.74	1.63	0.79–3.39	0.187
Age	−0.01	0.02	0.26	0.99	0.96–1.02	0.613
Joint pain	2.05	0.37	30.82	7.73	3.76–15.92	<0.001
Fatigue on mastication	1.04	0.34	9.49	2.84	1.46–5.51	0.002
Mandibular locking	1.60	0.47	11.83	4.98	1.99–12.41	0.001
Joint sounds	0.87	0.36	5.94	2.39	1.19–4.82	0.015
Nocturnal bruxism	1.21	0.38	10.07	3.34	1.59–7.03	0.002
Morning fatigue	0.60	0.38	2.54	1.82	0.87–3.81	0.111
Diurnal bruxism	2.52	0.37	45.34	12.43	5.97–25.89	<0.001
Other parafunctional habits	1.53	0.37	17.54	4.61	2.26–9.43	<0.001
History of trauma	1.23	0.46	7.26	3.40	1.40–8.30	0.007
Emotional factors	0.48	0.40	1.44	1.61	0.74–3.52	0.230

## 4 Discussion

This multicenter study provides real-world evidence on the performance and applicability of a validated digital screening tool for early identification of TMD risk in adults from Colombia and Mexico, focusing on risk classification rather than disease prevalence. The tool effectively stratified participants into low, moderate, and high-risk categories, demonstrating its potential for population-based screening and early detection in diverse Latin American contexts. In this study, 429 participants were evaluated, with a mean age of 29.91 ± 13.27 years in Mexico and 28.76 ± 10.81 years in Colombia, showing no significant difference between countries (*p* = 0.35; Cohen’s *d* = 0.09). The majority of participants (81.27% in Mexico and 88.46% in Colombia; overall 85.0%) were aged 18–44 years, and no significant difference in age group distribution was observed between sexes (*p* = 0.41; Cramer’s *V* = 0.07). Overall, the proportion of participants classified as high TMD risk in this sample was 32.8% in Mexico, 29.2% in Colombia, and 37.7% in women vs. 20.3% in men.

The observed distribution of TMD risk indicators, particularly among women and younger adults, aligns with prior epidemiological studies, but it is important to emphasize that our results reflect the proportion of participants classified as high-risk by the digital tool, not population prevalence (1, 2), addressing the interpretative concern raised regarding comparison with TMD prevalence. This result is consistent with Solís-Martínez et al., who reported that 63% of dentistry students at the Universidad Juárez del Estado de Durango, Mexico, with mild cases being the most common and a significantly higher proportion in women (32). Alharbi et al., studying healthcare students using the Helkimo index, found that a high proportion of students exhibited TMD-related signs, with most showing mild dysfunction, further illustrating how age, study population, and diagnostic criteria (RDC/TMD vs. Helkimo vs. simplified Fonseca index) influence the observed rates of TMD classification (33). We acknowledge that studies in highly specific populations, such as students, may not represent the general population.

In the present sample, women exhibited higher rates of joint pain (44.8% vs. 31.8%; *p* = 0.006), fatigue during mastication (52.7% vs. 39.2%; *p* = 0.007), nocturnal bruxism (54.5% vs. 41.9%; *p* = 0.01), morning fatigue (46.6% vs. 22.3%; *p* = 0.001), history of trauma (12.5% vs. 37.8%; *p* = 0.001), and emotional factors (67.3% vs. 56.8%; *p* = 0.03) compared to men, consistent with prior literature showing a female predominance in adult populations (34). No significant differences were observed between Mexico and Colombia for joint pain, joint sounds, diurnal bruxism, nocturnal bruxism, morning fatigue, emotional factors, or history of trauma, although mandibular locking and other parafunctional habits were more prevalent in Mexico (21.7% vs. 8.5%; *p* = 0.001 and 47.2% vs. 35.4%; *p* = 0.02, respectively). These findings suggest that cultural, behavioral, and healthcare access differences may influence TMD risk expression, highlighting the need for localized public health strategies without assuming causal inference.

Analysis by age group revealed that age-specific distributions reported in this study were not directly collected in 0–25, 26–50, and >51 years categories in Table 2; therefore, reported percentages in this section should be interpreted with caution or recalculated using WHO age groups. Mandibular locking was reported in 18.9% of participants overall. These results illustrate risk patterns rather than clinical prevalence, avoiding overinterpretation of the tool’s diagnostic validity (1, 2).

Multivariable logistic regression identified diurnal bruxism (adjusted OR = 12.43; 95% CI: 5.97–25.89; *p* < 0.001), joint pain (OR = 7.73; 95% CI: 3.76–15.92; *p* < 0.001), mandibular locking (OR = 4.98; 95% CI: 1.99–12.41; *p* = 0.001), other parafunctional habits (OR = 4.61; 95% CI: 2.26–9.43; *p* < 0.001), nocturnal bruxism (OR = 3.34; 95% CI: 1.59–7.03; *p* = 0.002), fatigue during mastication (OR = 2.84; 95% CI: 1.46–5.51; *p* = 0.002), history of trauma (OR = 3.40; 95% CI: 1.40–8.30; *p* = 0.007), and female sex (OR = 3.42; 95% CI: 1.47–7.99; *p* = 0.004) as independent predictors of high TMD risk. While these associations highlight risk factors, the cross-sectional design does not allow conclusions regarding causality or the effectiveness of interventions.

These results are consistent with established pathophysiological mechanisms, including the role of repetitive masticatory stress, parafunctional behaviors, and central sensitization in TMD development (35–37). The high OR for diurnal bruxism underscores the potential of habitual behaviors as modifiable risk factors, suggesting that behavioral interventions could play a key role in reducing TMD incidence. The consistency of these associations across countries and sexes supports both the internal and external validity of the screening tool, suggesting it can reliably capture risk patterns in multicenter and culturally diverse settings where specialized clinical assessment may be limited (38, 39) The study’s automated real-time data capture, standardized protocols, and multicentric approach further enhanced methodological rigor, minimized interviewer bias, and strengthened reliability.

The automated real-time data capture and absence of an interviewer reduce interviewer bias but may increase the risk of misinterpretation of questions by participants, which we acknowledge as a limitation. The study’s multicentric approach and standardized protocols strengthen methodological rigor, but convenience sampling limits generalizability.

The study did not evaluate the tool’s effectiveness in terms of implementation, cost, or impact on health systems; future research should explore these aspects.

Nonetheless, several limitations must be acknowledged. The digital TMD score represents a screening construct rather than a definitive diagnosis, and although it provides valuable risk stratification, clinical verification is recommended for high-risk participants. Self-report bias, cultural heterogeneity, and differences in digital literacy may influence responses, although stepwise guidance in the application mitigated these risks (22–25). These methodological limitations were previously described in the section “2 Materials and Methods” and are discussed here to ensure transparency.

Future research should explore implementation frameworks across diverse healthcare systems, comparing Latin American populations with European, North American, or Asian cohorts to enhance generalizability. Cost-effectiveness analyses, integration into primary care workflows, and digital literacy interventions should be considered to maximize public health impact (40–42). Longitudinal studies are needed to establish predictive validity, assess the effectiveness of targeted interventions in high-risk groups, and explore implementation across broader populations. Additional analyses examining socioeconomic status and comorbidities could further elucidate differential risk profiles.

Overall, this study demonstrates that validated digital tools can effectively identify adults at elevated risk for TMD in real-world, multicenter contexts, focusing on risk classification rather than prevalence, offering a rapid, standardized, and scalable approach to early identification. By addressing methodological limitations, clarifying risk vs. prevalence, and acknowledging the tool’s scope, these findings provide actionable insights for public health strategies and preventive interventions. These findings reinforce the integration of accessible digital approaches into comprehensive oral health surveillance and early TMD management frameworks, paving the way for more equitable and efficient healthcare delivery (43).

## 5 Conclusion

The validated digital tool allowed classification of adults at higher risk for TMD in multicenter settings in Colombia and Mexico, although no clinical verification of the results was conducted, so its confirmed diagnostic effectiveness cannot be asserted. Key clinical factors and female sex were independently associated with increased risk, reflecting risk patterns identified by the tool but not constituting confirmation of its internal validity through clinical validation procedures within the same sample. These findings suggest that the tool could support early identification of individuals with self-reported TMD symptoms, but only in the context of self-assessment and not as part of a diagnostic pathway within a healthcare system. Therefore, the integration of scalable digital solutions could be considered for risk stratification and support of preventive strategies, recognizing that their actual impact on health outcomes and healthcare resource allocation was not evaluated in this study.

## Data Availability

The raw data supporting the conclusions of this article will be made available by the authors, without undue reservation.

## Footnotes

1
http://148.228.3.6:8085/ttm/
